# (Un)safety of LGBT+ asylum seekers in asylum accommodations in Germany

**DOI:** 10.1093/eurpub/ckac130.169

**Published:** 2022-10-25

**Authors:** F Gartner, M Giacomozzi, A Helberg-Proctor

**Affiliations:** 1 Faculty of Health, Medicine and Life Sciences, Maastricht University, Maastricht, Netherlands; 2 Treat it Queer Foundation, Maastricht, Netherlands; 3 Life Sciences & Society Lab, KU Leuven, Leuven, Belgium

## Abstract

LGBT+ asylum seekers face multiple challenges in their countries of origin as well as in their host countries. Violence and structural discrimination against this community are common and affect their whole asylum process. Violence and structural discrimination based on sexual orientation and gender identity stress the needs of LGBT+ asylum seekers in particular in regards to reception accommodations. In this study we investigated factors that contribute to the (un)safety of reception accommodations for LGBT+ asylum seekers in Germany. Qualitative semi-structured interviews were conducted with 11 participants from two groups, namely, professionals, and former and current LGBT+ asylum seekers. Thematic analysis was used to analyze the data and revealed multiple factors influencing the safety of LGBT+ asylum seekers in accommodations. These were clustered according to the used frameworks in the categories individual level, physical and social environment, and policies. The factors for (un) safety included amongst others interpersonal violence based on sexual orientation and gender identity, gender-neutral sanitary areas or lockable rooms, community support, and policies that govern where asylum seekers are accommodated, and which protection measures are set up in accommodations. Applying an intersectional lens, transgender asylum seekers were described as more vulnerable than other LGBT+ individuals. The analysis concluded that binding policies are necessary to guarantee safer accommodations for LGBT+ asylum seekers in Germany. Besides, the social cisheteronormative structures that manifest in discrimination of LGBT+ asylum seekers must be structurally deconstructed by, among others, training staff on LGBT+ needs and increase inclusivity among the asylum seeker community and in the host country.

**Key messages:**

- In Germany, the European country with the highest number of refugees, LGBT+ asylum seekers face many challenges related to their sexual orientation and gender identity.
- Several factors interplay in the (un)safety of accommodations for LGBT+ asylum seekers.

